# Sense of Resistance for a Cursor Moved by User’s Keystrokes

**DOI:** 10.3389/fpsyg.2021.652781

**Published:** 2021-04-29

**Authors:** Takahiro Kawabe, Yusuke Ujitoko, Takumi Yokosaka, Scinob Kuroki

**Affiliations:** NTT Communication Science Laboratories, Nippon Telegraph and Telephone Corporation, Atsugi, Japan

**Keywords:** sense of resistance, pseudo-haptic feedback, keystroke, illusion, cross-modal interaction

## Abstract

Haptic sensation of a material can be modulated by its visual appearance. A technique that utilizes this visual-haptic interaction is called as pseudo-haptic feedback. Conventional studies have investigated pseudo-haptic feedback in situations, wherein a user manipulated a virtual object using a computer mouse, a force-feedback device, etc. The present study investigated whether and how it was possible to offer pseudo-haptic feedback to a user who manipulated a virtual object using keystrokes. Participants moved a cursor toward a destination by pressing a key. While the cursor was moving, the cursor was temporarily slowed down on a square area of the screen. The participants’ task was to report, on a five-point scale, how much resistance they felt to the cursor’s movement. In addition to the basic speed of the cursor, the ratio of the basic speed to the speed within the square area was varied. In Experiment 1, we found that these two factors interacted significantly with each other, but further analysis showed that the cursor speed within the square area was the most important determinant of perceived resistance. In Experiment 2, consistent with the results of the previous experiment, it was found that the cursor movement outside of the square area was not required to generate the sense of resistance. Counterintuitively, in Experiment 3, the sense of resistance was apparent even without user’s keystrokes. We discuss how the sense of resistance for a cursor moved by keystrokes can be triggered visually, but interpreted by the brain as a haptic impression.

## Introduction

One of the important goals of the brain’s perceptual system is to generate coherent perceptual representations of the world on the basis of sensory inputs. Importantly, sensory modalities are not independent of each other (Shimojo and Shams, 2001), so the representation generated by the brain is in general cross-modal (or multimodal). Based on the cross-modal interaction between haptics and vision, it is known that haptic experience is strongly modulated by visual inputs (Rock and Victor, 1964; Singer and Day, 1969; Lederman and Abbott, 1981). More recent studies have shown that haptic experience is dependent on the optimal integration of haptic and visual inputs (Ernst and Banks, 2002; Hillis et al., 2002).

Pseudo-haptic feedback (Lécuyer, 2009) is an information presentation technique that, from an engineering point of view, aims to modulate haptic sensations by changing visual appearance. In the typical situation of pseudo-haptic feedback, users manipulate a virtual object in a visual display by controlling haptic devices. Changing the visual appearance of the visual object often provides illusory haptic sensations to the users manipulating the object. For example, previous studies have shown that pseudo-haptic feedback can modulate the perception of virtual spring compliance (Lécuyer et al., 2000), friction (Lécuyer et al., 2000; Ujitoko et al., 2019a), object edge angle (Ban et al., 2012), mass (Dominjon et al., 2005; Issartel et al., 2015; Yu and Bowman, 2020), weight (Brewster et al., 2019), and texture (Ujitoko et al., 2019b; Sato et al., 2020). A key principle of pseudo-haptic feedback is the spatio-temporal dissociation of vision, haptics, proprioception, and motor control. In particular, the ratio of the amplitude of the hand motion (i.e., control) to the amplitude of the cursor motion (i.e., display), the so-called C/D ratio (Lécuyer et al., 2004; Dominjon et al., 2005), plays an important role in pseudo-haptic feedback.

So far, pseudo-haptic feedback has been studied in situations, where a user manipulates a virtual object (e.g., a cursor) by operating a computer mouse (Lécuyer et al., 2004; Dominjon et al., 2005; Kumar et al., 2017), tablet PCs (Ujitoko et al., 2015; Costes et al., 2019), pen devices (Ujitoko et al., 2019a,b), virtual hands (Sato et al., 2020), real objects (Brewster et al., 2019), and mixed reality technologies (Ban et al., 2012; Issartel et al., 2015; Kawabe, 2020). The use of these devices involves hand movements that are more or less consistent in direction with the movement of the virtual object. For example, if a user makes a hand movement to the right in the real world, the virtual object will move to the right on the screen. The magnitude of the movement of the virtual object is controlled based on a predetermined C/D ratio.

On the other hand, it is also possible to move a virtual object without making directional hand movements that coincide with the movement of the virtual object. A typical such device is the game controller. In a video game application, a user employs the direction pad of the game controller to control a virtual object/character. By pressing down on a part of the direction pad, the virtual object/character moves in a two- or three-dimensional virtual world. A key point is that the direction of movement of keystrokes on the direction pad does not coincide with the direction of movement of the virtual object; when a key is pressed down, the virtual object moves in the two-dimensional and/or three-dimensional directions in the display. Another key point is that the distance moved by the virtual character depends on the length of time the user holds down the assigned key, whereas in the typical situation of pseudo-haptic feedback research, the movement distance of the virtual object depends on how much the user moves her/his hand. Thus, the control of the virtual character based on keystrokes depends on different information from that used in the control based on hand movements that have been used in previous pseudo-haptic studies. Because of this, it was unclear whether we could provide pseudo-haptic feedback to a user who controlled the virtual object using keystrokes. A previous study (Argelaguet et al., 2013) employed a change in color of the cursor caused by the user pressing a mouse button as additional visual feedback in judging pseudo-haptic elasticity. However, until now little systematic knowledge has been acquired on the role of keystrokes in pseudo-haptic feedback.

In the present study, we attempted to specify stimulus conditions that would provide a sense of resistance to a user who manipulated a cursor only using keystrokes. In previous studies, pseudo-haptic feedback has been discussed in terms of physical properties such as friction (Lécuyer et al., 2000; Ujitoko et al., 2019a), mass (Dominjon et al., 2005; Issartel et al., 2015; Yu and Bowman, 2020), and viscosity (Costes et al., 2019). In the present study, experimental participants manipulated the cursor by keystrokes, and the stimuli observed by the participants consisted of simple geometric patterns such as squares and circles. Therefore, there was no sufficient *a priori* reason to attribute pseudo-haptic impressions, which would be obtained in the experimental situation of the present study, to a certain type of the physical properties. We consider the sense of resistance as a higher-order component of the pseudo-haptic impressions that have been discussed in terms of the physical properties as described above. By investigating the sense of resistance, we surmise that it is not necessary to assume any relationship between pseudo-haptic impressions and physical properties. Hence, we decided to ask the participants to report their sense of resistance.

In the experiments reported in this study, the movement of the cursor, initiated by keystrokes, was slowed down when the cursor entered a square area in the center of the display. For the cursor speed, two factors were varied: the basic speed and the speed ratio. The basic speed was the cursor speed when outside the square area. The speed ratio was the ratio of the cursor speed inside the square area to the cursor speed outside the square area. In Experiment 1, we examined whether and how the two factors of cursor speed affected the sense of resistance. In Experiment 2, we tested whether the pattern of speed change along the cursor path was important in determining the sense of resistance. In Experiment 3, we examined whether continuous operation of a key is a necessary factor for resistance. Based on the results obtained, it is concluded that the sense of resistance for the cursor moved by keystrokes originates from visual effects, and at the same time, the cross-modal transfer (Biocca et al., 2001) causes haptic sensations related to the sense of resistance.

## Experiment 1

### Purpose

The purpose of the experiment was to examine whether the participants could feel resistance to the cursor moving with their keystrokes. In the experiment, the participants controlled the movement of the cursor by pressing and holding down a key on the keyboard. The cursor was operated at three basic speeds (actually 2, 4, and 6 pixels/display frame of the computer monitor). When the cursor passed through the central square area, the speed was reduced with the three levels of speed ratio. The effect of speed ratio and basic speed on the degree of resistance was tested.

### Method

#### Participants

Two hundred and two people (101 females) participated in this experiment. Their mean age was 39.73 (SD 11.33). A Japanese crowdsourcing research company recruited them online and paid for their participation. They were unaware of the specific purpose of the experiment. Ethical approval for this study was obtained from the Ethics Committee at Nippon Telegraph and Telephone Corporation (Approval number: R02-009 by NTT Communication Science Laboratories Ethics Committee). The experiments were conducted according to principles that have their origin in the Declaration of Helsinki. Written informed consent was obtained from all observers in this study.

#### Apparatus

The experiment for this study was carried out on a personal computer (PC) provided by the participant. Since the experimental script runs only on a PC, a smartphone or a tablet PC could not be used. The viewing distance and the screen size were not controlled because their effect was not evident in the preliminary observation as long as the user used the PC under normal use conditions.

#### Stimuli

**Figure 1:**
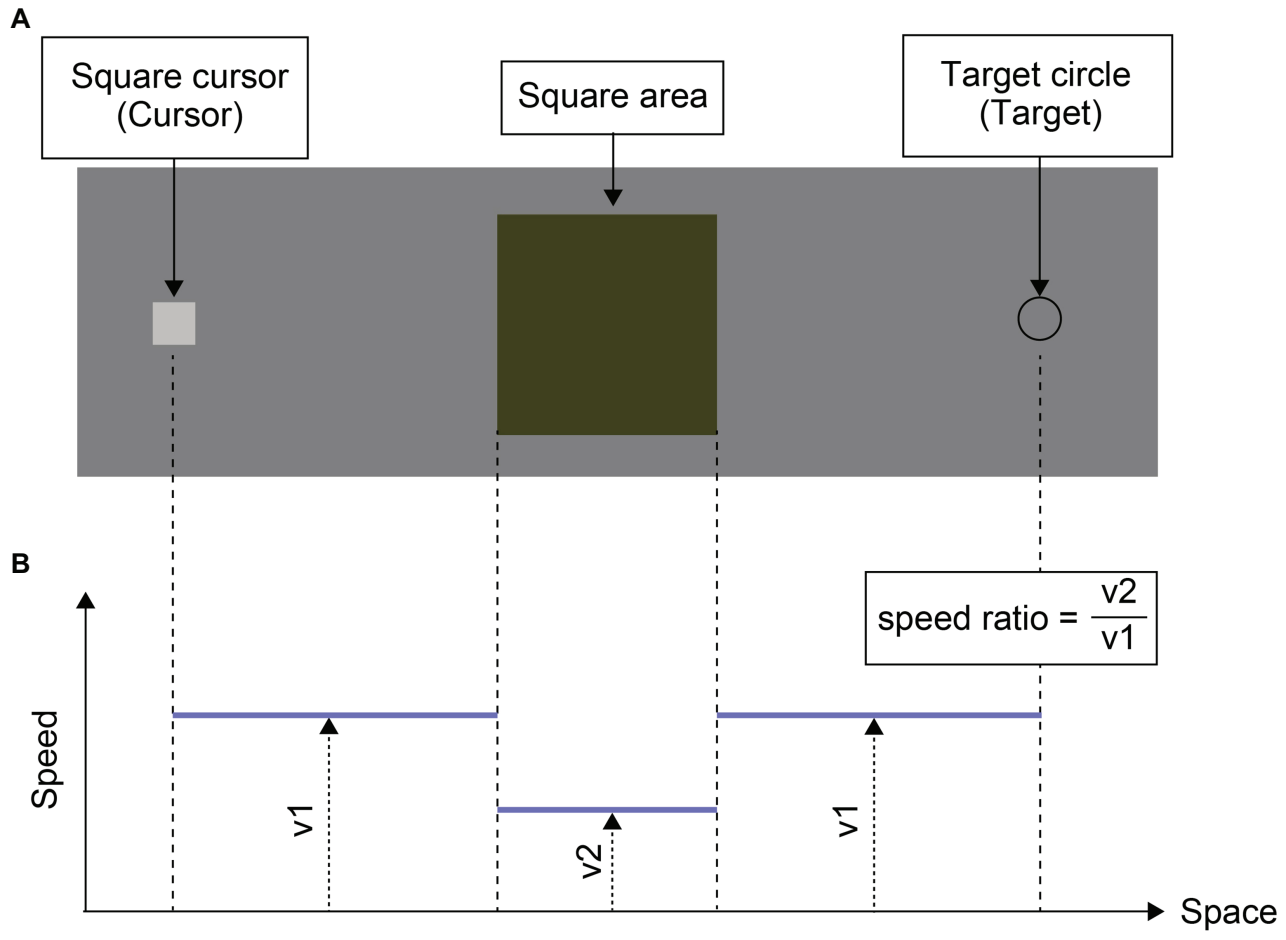
**(A)** Snapshot of the stimulus with the description of the components. Arrows and text are used to describe the stimulus components and are not shown in the actual experiment. **(B)** Schematic graph showing the change in cursor speed over the cursor path. As shown in the inset, the speed ratio is defined as v2/v1. Vertical dashed lines show the correspondence between the spatial positions in **(A)** and **(B)**.

As shown in Figure 1, the stimuli consisted of a square cursor [16 x 16 pixels, with RGB values (192, 192, 192)], a square area [120 x 120 pixels, with RGB values (64, 64, 0)], a target circle [16 pixels in diameter with RGB values of the contour (32, 32, and 32)], and a uniform background with RGB values (128, 128, 128). The initial locations of the cursor (and thus the target), and hence the direction of travel of the cursor from left to right or right to left, were randomly determined from trial to trial. When the cursor appeared 200 pixels to the left of the left edge of the square area, the target appeared 200 pixels to the right of the right edge of the square area. On the other hand, when the cursor appeared 200 pixels to the right of the right edge of the square area, the target appeared 200 pixels to the left of the left edge of the square area. The cursor moved toward the target only when the participant held down an assigned key (i.e., the M key on the computer keyboard). Because the square area was centered in the display, the cursor went across the square area to reach the target. Outside the square area, the cursor moved at a speed of 2, 4, or 6 pixels per frame. This speed is called as “basic speed.” The basic speed was set at the three levels because, according to preliminary observations, it was not so easy to perceptually track the cursor moving at higher speeds, which would result in uncomfortable cursor manipulations for users. Within the square area, the cursor moved at the basic speeds of 0.25x, 0.5x, and 1x the basic speed. This modulation of velocity in the square area is called as the “speed ratio.”

#### Procedure

At the beginning of the experiment, the participants were presented with written instructions that described the situation and tasks of the experiment. After reading this, the participant pressed the space key on the computer keyboard to start the experiment. The participant’s task was to hold down the M key and so allow the cursor to move toward the target. The stimulus images disappeared after the cursor reached the target. The participants then assessed how much resistance they felt while the cursor was moving over the square area. They reported the evaluation on a five-point scale by pressing the assigned keys, wherein five represented the highest resistance. After reporting their impression, the next trial began. Each experimental condition was repeated five times. Thus, each participant performed 45 trials (three basic speed conditions × three speed ratios × five iterations) in a pseudo-random order.

### Results

**Figure 2:**
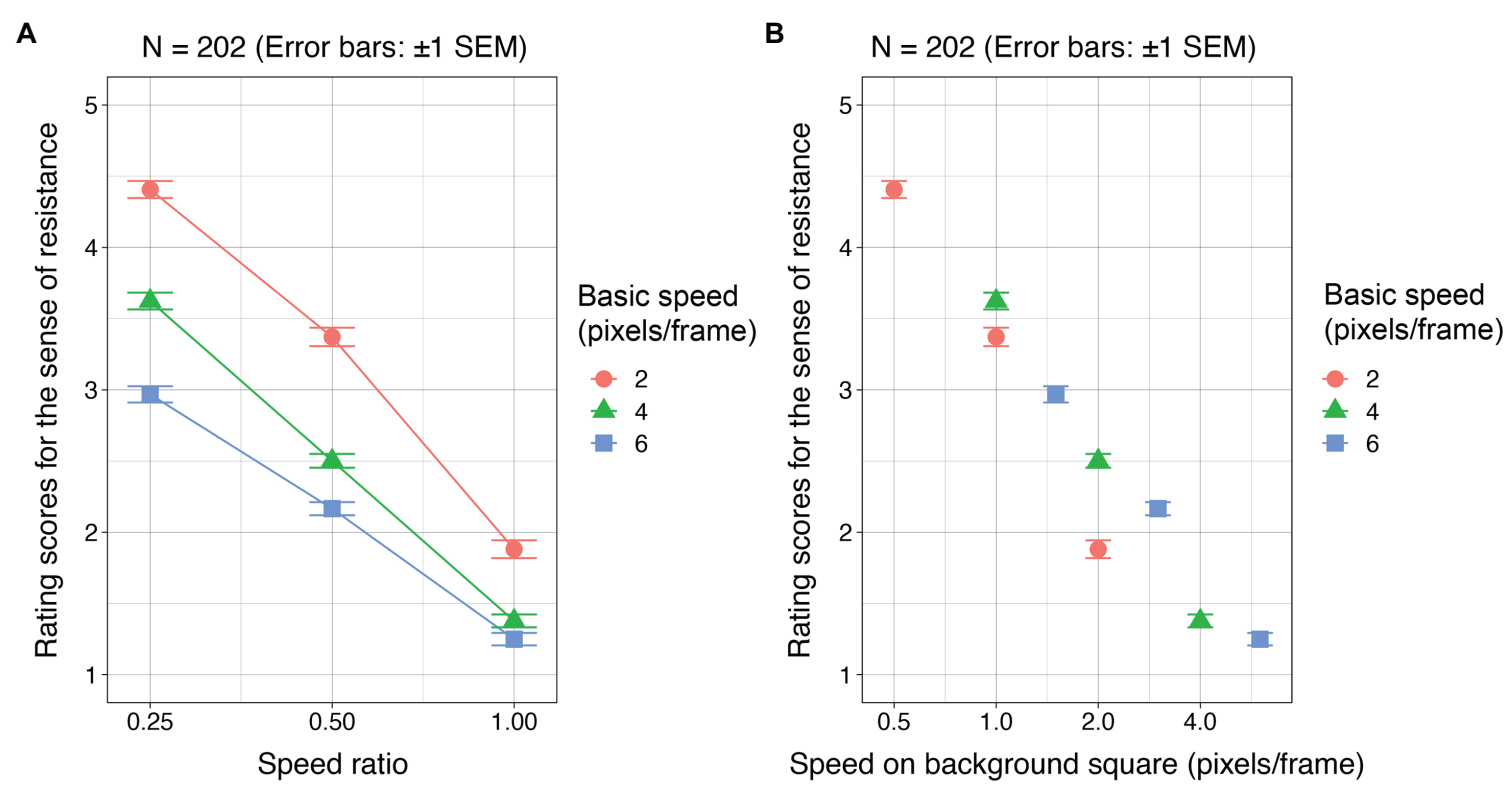
Experiment 1 results. **(A)** For each of the basic speeds, rating scores for the sense of resistance are plotted as a function of the speed ratio. **(B)** The rating scores are plotted as a function of the cursor speed on the square area.

For each condition, rating scores were averaged for each participant. Mean rating scores across the participants are shown in Figure 2A. By using “vegan” (Oksanen et al., 2020) and “EcolUtils” (Salazar, 2020) packages of R (R Core Team, 2020), a two-way permutation analysis of variance (Anderson, 2001; Anderson and Walsh, 2013) was conducted with the basic speed and speed ratios as within-subject factors. The main effect of the basic speed was significant (*F* = 198.58, *p* < 0.001, *r*^2^ = 0.08). The main effect of the speed ratio was also significant (*F* = 1113.16, *p* < 0.001, *r*^2^ = 0.49). The interaction between them was significant (*F* = 19.45, *p* < 0.001, *r*^2^ = 0.017). All *post-hoc t*-tests on the basis of the significant interaction reached significance (*p* < 0.05 with the Bonferroni correction).

Although the interaction between the basic speed and speed ratio was clear, the rating scores might simply depend on the cursor speed on the square area. Figure 2B plots the rating score as a function of the cursor speed on the square area. We can see the rating score clearly depends on the cursor speed. By using data from all participants, we performed a linear regression analysis and found the regression to be significant [*Adjusted r*^2^ = 0.47, *F*(1, 1825) = 1,662, *p* < 0.001]. Furthermore, the cursor speed on the square area significantly contributed to the regression (*t* = −40.77, *p* < 0.001).

As a result, it was found that the cursor speed in the square area was critical to generating the sense of resistance. As we varied the basic speed and the speed ratio as experimental factors, the cursor speed within the square area also varied with the changes. As shown in Figure 2B, and by the regression analysis, the rating scores can be well described by a single parameter, the cursor speed when in the square area. In a sense, the results are consistent with the previous results (Lécuyer et al., 2004; Dominjon et al., 2005), showing that the C/D ratio regulates the pseudo-haptic feedback. In the current experiment, since the cursor movement was controlled by pressing and holding down a key, the speed of hand movement was kept almost constant. Therefore, for the C/D ratio, C was a constant, and thus, the C/D ratio in this experiment depended only on D, i.e., the cursor speed.

It was still unclear whether the speed ratio had any effect on the sense of resistance because the modulation of the speed ratio was not experimentally dissociated from the variation of the cursor speed in the square. In the next experiment, we attempted to clarify this issue.

## Experiment 2

### Purpose

The purpose of the experiment was to check whether a sense of resistance could be caused even when the cursor did not move outside the square area and thus no speed ratio could be obtained. The results of the previous experiment showed that the cursor speed within the square area was critical to regulating the sense of resistance, indicating that the movement path outside the square area might possibly play a minor role in determining the sense of resistance. In the current experiment, we completely or partially removed the path outside the square area to see the effect of the path on the sense of resistance.

### Method

#### Participants

Two hundred and three people (101 females), who had not participated in Experiment 1, participated in this experiment. Their mean age was 40.25 (SD 11.25).

#### Stimuli

**Figure 3:**
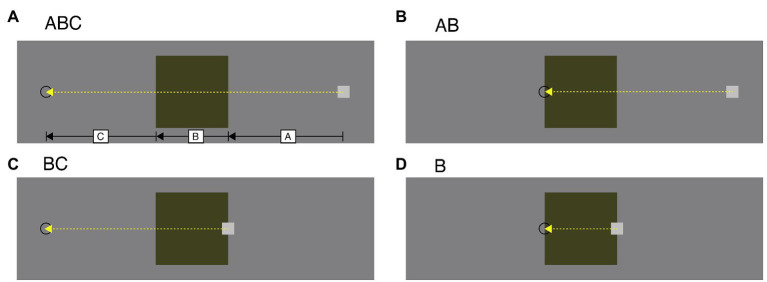
Schematic explanations of the movement path of the square cursor in the Experiment 2 stimuli. The labels “A,” “B,” and “C” as well as the lines and arrows are used to describe the path patterns and were not presented in the actual experiment. Each panel shows (A) ABC condition, (B) AB condition, (C) BC condition, and (D) B condition.

The stimuli were identical to those used in Experiment 1 except for the following. We tested four conditions (in other words, there are three new conditions in addition to the previous one) on the movement path of the cursor. As in the Experiment 1, the appearance locations of the cursor (and thus the target) were randomly determined from trial to trial. In the “ABC” condition (Figure 3A), as in Experiment 1, when the cursor appeared 200 pixels to the right of the right edge of the square area, it moved through the square area to the target presented 200 pixels to the left of the left of the square area. When the cursor appeared 200 pixels to the left of the left edge of the square area, it moved through the square area to the target presented 200 pixels to the right of the right of the square area. In the “AB” condition (Figure 3B), when the cursor appeared 200 pixels to the right of the right edge of the square area, it moved through the square area to the target presented at the left edge of the square area. When the cursor appeared 200 pixels to the left of the left edge of the square area, it moved through the square area to the target at the right edge of the square area. In the “BC” condition (Figure 3C), when the cursor appeared at the right edge of the square area, it moved through the square area to the target presented 200 pixels to the left of the left edge of the square area. When the cursor appeared at the left edge of the square area, it moved through the square area to the target presented 200 pixels to the right of the right edge of the square area. In the “B” condition (Figure 3D), when the cursor appeared at the right edge of the square area, it moved through the square area to the target presented at the left of the square area. When the cursor appeared at the left edge of the square area, it moved through the square area to the target presented at the right edge of the square area. The basic speed was kept constant at 4 pixels/frame for all trials. In each trial, the speed ratio was randomly selected from among three candidates of 0.25x, 0.5x, and 1x. Since we used only one level of the basic speed, the cursor speed in the square area was 1, 2, and 4 pixels/frame.

#### Procedure

The procedure was identical to that used in Experiment 1 except for the following. Each participant performed 60 trials (four movement paths × three speed ratios × five iterations) in a pseudo-random order.

### Results and Discussion

**Figure 4:**
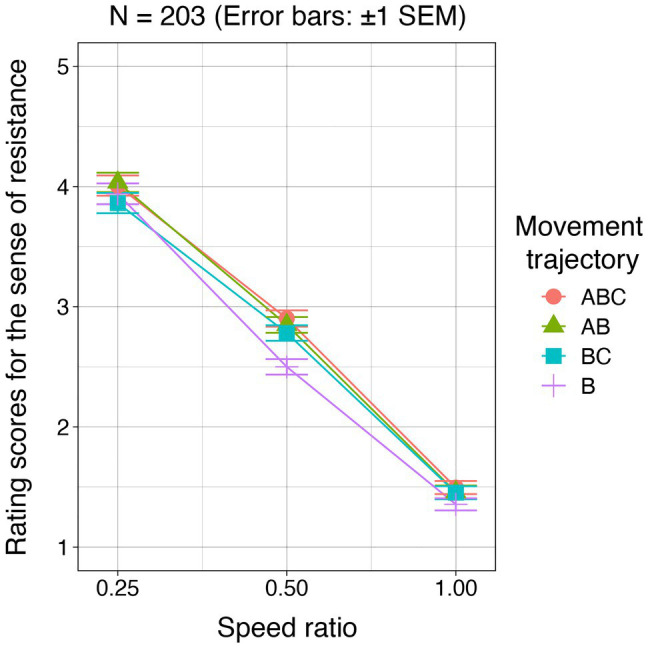
Experiment 2 results. For each of the movement paths, the rating scores for the sense of resistance are plotted as a function of the speed ratio.

For each condition, rating scores were averaged for each participant. Mean rating scores across the participants are shown in Figure 4. By using individual mean rating scores, a two-way permutation analysis of variance was conducted with the movement trajectories and speed ratios as within-subject factors. The main effect of the movement trajectories was significant (*F* = 5.28, *p* = 0.002, *r*^2^ = 0.003). However, the multiple comparison tests with the Bonferroni correction did not show any significant difference between any two of the movement trajectories (*p* > 0.05). The main effect of the speed ratio was significant (*F* = 1184.91, *p* < 0.001, *r*^2^ = 0.49). The multiple comparison test showed that there were significant differences between any two of the speed ratios (*p* = 0.003). Interaction between the two factors was not significant (*F* = 1.55, *p* = 0.13).

The results indicate that the presence/absence of motion paths outside the square area played a minor role in generating the sense of resistance. Because the effect of the speed ratios was significant, the sense of resistance is likely determined on the basis of the cursor speed in the square area, which is consistent with Experiment 1. Thus, the effect of the speed ratio on the magnitude of the sense of resistance can be attributed to the cursor speed in the square area.

## Experiment 3

### Purpose

The purpose of this experiment was to explore whether the participant’s keystrokes played an essential role in generating the sense of resistance. We tested the following three conditions: in a “hold-down” condition, as in Experiment 1, the participants kept holding down a key to control the cursor movement. In an “automatic” condition, the participants observed the cursor movement without pressing a key. In a “release” condition, the participants pressed and then released a key to start the cursor movement. If the participant’s action (i.e., pressing a key) was necessary for generating the sense of resistance, the rating scores for the automatic condition would be lower than those for the other conditions. Moreover, if generating the sense of resistance required the participants’ continued involvement by their action, the rating scores for the hold-down condition would be greater than that for the release condition.

### Method

#### Participants

Two hundred and three people (101 females), who had not participated in Experiments 1 and 2, participated in this experiment. Their mean age was 40.11 (SD10.91).

#### Stimuli

The stimuli were identical to those used in Experiment 1 except for the following. We tested three starting conditions (in other words, there are two new conditions in addition to the previous one.) on how the cursor started to move. First, in the “Hold-down” condition, as in Experiment 1, the square cursor moved toward the target only when the participant kept holding down the M key. Second, in the “Automatic” condition, the participants watched the cursor that automatically moved toward the target. Specifically, in the first trial, the cursor moved 2 s after the participant started the session by pressing the spacebar on the keyboard. In the second and subsequent trials, the cursor began to move 2 s after the participant reported a judgment about the sense of resistance for a previous trial. Third, in the “Release” condition, the cursor moved immediately after the finger was released from the M key. The basic velocity was kept constant at 4 pixels/frame. As in previous experiments, three levels of speed ratio (0.25x, 0.5x, and 1x) were used.

#### Procedure

In each trial, an instruction for starting the cursor movement was presented above the stimuli. In the “Hold-down” condition, the statement “Hold down the M key and watch the cursor move” was presented. In the “Automatic” condition, the statement “Watch the cursor without pressing any keys” was shown. In the Release condition, the statement “Release the M key to move the cursor and observe cursor movement” was presented. Following the instructions, the participants moved and/or observed the cursor. Other aspects of the task were the same as those used in Experiment 1, with the following exceptions. Each observer performed 45 trials [three starting conditions (Hold-down, Automatic, and Release) × three speed ratios × five iterations] in a pseudo-random order.

### Results and Discussion

**Figure 5:**
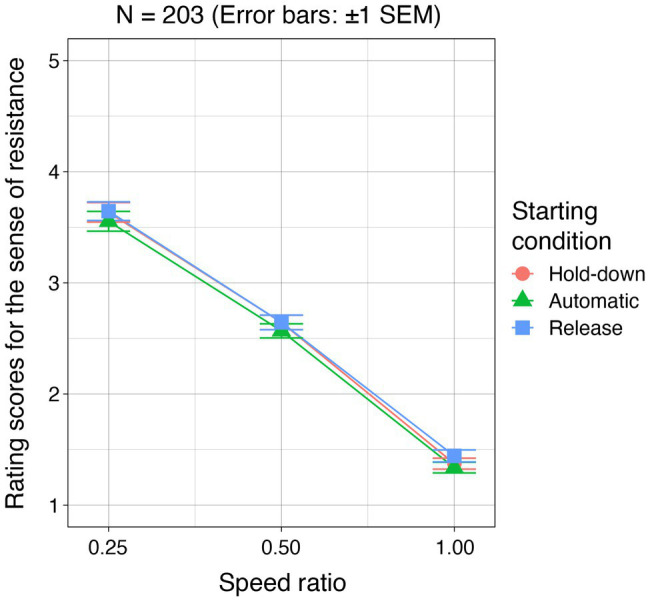
Experiment 3 results. For each starting condition, rating scores for the sense of resistance are plotted as a function of the speed ratio.

For each condition, rating scores were averaged for each participant. Mean rating scores across the participants are shown in Figure 5. By using individual mean rating scores, a two-way permutation analysis of variance was conducted with the starting conditions and speed ratios as within-subject factors. The main effect of the starting conditions was not significant (*F* = 1.52, *p* = 0.205, *r*^2^ = 0.0009). The main effect of the speed ratio was significant (*F* = 770.05, *p* < 0.001, *r*^2^ = 0.45). The multiple comparison test showed that there were significant differences between any combination of the speed ratios (*p* = 0.003). The interaction between them was not significant (*F* = 0.16, *p* = 0.977, *r*^2^ = 0.0002).

The results that the main effect of the starting condition was not significant, which indicates that the participants’ action is not a necessary condition for the generation of the sense of resistance for a cursor moved by user’s keystrokes, at least under the conditions we investigated. The sense of resistance is likely a visual phenomenon and the visually-caused sensation is interpreted as the sensation for a moving object under key control.

## General Discussion

The findings of this study can be summarized as follows. When the cursor, controlled by the participant’s keystrokes, slowed down, the participant could sense resistance. The cursor speed itself, rather than the speed ratio on the cursor path, was an important indication of resistance. The participant’s keystrokes were not a necessary factor in generating a sense of resistance.

The effect of cursor speed had a critical influence on the sense of resistance. As shown in Figure 2B, the rating scores of the sense of resistance within the square area decreased linearly as a function of the cursor speed. On the other hand, it remains an open question whether the linear relationship between the sense of resistance and the cursor speed is maintained when the range of the cursor speed is extended. For example, if there are extreme cursor speeds in the stimulus set, the linear relationship is expected to be easily disturbed. Therefore, when designing the sense of resistance in an actual GUI, engineers need to pay attention to the range of cursor speed to provide the desired level of the sense of resistance to the cursor. Since we used a relatively low level of the basic speed in this study, we cannot predict the pattern of the resistance sensation when a higher basic speed is employed. This is a limitation of the present study.

It should be noted that our experimental setup does not guarantee that all participants experienced comparable cursor speeds. We controlled the cursor speed in the unit of pixels/frame (i.e., how far the cursor moved in pixels between two consecutive frames). Thus, the actual speed (e.g., in units of cm/s) varied depending on the spatial and temporal resolutions of the display the participants used for their experiments. In addition, since we controlled neither the size of the display nor the observation distance, the retinal speed (i.e., in deg./s) was likely to have varied among participants. Despite the potential diversity of displays used in the experiment, the effect of cursor speed on resistance was robust. This suggests that each participant may have judged the resistance based on the relative cursor speed in a given stimulus set. Future research is needed to further clarify possible interactions between absolute and relative cursor speeds, controlling the spatio-temporal resolution of the display, display size, and observation distance.

No effect of participant’s action (i.e., keystrokes) was observed. In other words, whether the participants manually controlled the cursor or not had no effect on the magnitude of the sense of resistance. As discussed in Experiment 1, since action involving key control is almost constant, C (i.e., the hand speed) will also be constant when calculating the C/D ratio. Ultimately, the C/D ratio depends on D (i.e., the cursor speed). We speculate that the reason why we did not observe the effect of key control action on the sense of resistance was simply that the magnitude of motor action and/or haptic stimulation involving keystrokes was smaller than other types of motor action that were tested in the previous studies on pseudo-haptic feedback. The relatively smaller magnitude of motor action compared with visual movements perhaps reduced the contribution of motor action to the cross-modal integration causing the sense of resistance. Future studies should examine whether the effect of motor action on the sense of resistance is observed when the cursor is moved by other motor actions, such as mouse movements, rather than key presses.

Although the difference in motor action did not contribute to the variation in the amount of the sense of resistance, this does not necessarily mean that the sense of resistance observed in this study is a purely visual representation; it is possible that haptic and/or motor components may also be included in the representation of the sense of resistance. When the authors performed the task in a preliminary experiment, they felt a non-visual (or haptic) sense of resistance when manually operating the cursor. This informal experience is consistent with the interpretation based on cross-modal transfer (Biocca et al., 2001). In Biocca et al. (2001), the participants were asked to manipulate objects in the virtual world without receiving force or haptic feedback. Interestingly, the participants reported that they experienced physical force even when neither force nor haptic feedback was given. The authors argued that the illusory haptic sensation was due to cross-modal transfer or synesthesia (Baron-Cohen, 1996). As is well known, each sensory modality in the brain is not independent of the others (Shimojo and Shams, 2001). The modalities interact with each other to generate cross-modal representations. From this perspective, it is reasonable to assume that visual resistance is transferred to haptic and/or sensorimotor sensations, which are used to generate internally a cross-modal model of the world. Thus, it may be possible to clarify whether or not the sense of resistance forms a cross-modal representation, for example, by examining the brain regions involved in this phenomenon.

One previous study (Watanabe, 2013) reported that pseudo-collision impression was induced by the sudden slowdown of a moving object, which occurred when the object and a mouse cursor were spatially coincident with each other. At first glance, the results of the previous study and the present study appear to be inconsistent. In the present study, the cursor speed after slowing down was important for the magnitude of the sense of resistance, whereas, in Watanabe (2013), the object speed after slowing down was not the only factor determining the pseudo-collision impression. We suggest, however, that the difference in the pattern of results may reflect the differences in procedures and stimuli between the previous study and the present study. In the previous study, the participants were asked to report the magnitude of pseudo-collision impression. In the typical case of object collision, the speed of the moving object is expected to slow down after the collision, so it is likely that the relative movement speed was used as a cue to determining the pseudo-collision impression. In the present study, on the other hand, we asked the participants to report the sense of resistance when the cursor passed over the square area. The task of the current study was possibly made feasible by focusing on the cursor speed within the square area only. The difference in motion patterns on which the participants relied to perform the task is possibly the cause of the difference between Watanabe (2013) and present studies. Although in Watanabe (2013) the pseudo-collision impression was not induced when no speed reduction was applied to the moving object, the sense of resistance was generated in the present study even when the cursor speed did not change along the cursor path as long as it was sufficiently low, as observed in the lowest basic speed condition with the speed ratio of 1. Thus, we suggest that the pseudo-collision impression is likely a perceptual phenomenon different from the sense of resistance.

To sum up, is the sense of resistance shown in this study different from the pseudo-haptic sensations that have been repeatedly shown in previous studies? We believe that they are essentially the same thing. However, the contribution of this study is that we showed that keystrokes can be used to generate pseudo-haptic sensation. We are not saying that the pseudo-haptic resistance is specific to keystrokes. It is therefore worth investigating the difference in the strength of the pseudo-haptic resistance effects among different input devices such as a computer mouse, a pen-type device, and a force-sensing device. If the phenomenon we discovered is based on the cross-modal transfer, the similar pseudo-haptic phenomenon can be observed among input devices.

By expanding the variations of pseudo-haptic sensations that were caused by users’ keystrokes, it will be possible to give users richer keystroke experiences. For example, assume a situation, where a user controls a drone by using a direction pad and the drone unintentionally slows down due to some pre-built programs, even though in reality there is no impediment to the drone’s movement and it should not slow down. Under that scenario, based on the outcome of the present study, it is expected that the user will feel resistance. By using the pseudo-haptic effect, it may be possible to give the user the impression that a drone flying in the sky is passing through a virtual “viscous” space, or that the drone is creating friction with an invisible object.

## Data Availability Statement

The raw data supporting the conclusions of this article will be made available by the authors, without undue reservation.

## Ethics Statement

The studies involving human participants were reviewed and approved by the NTT Communication Science Laboratories Ethics Committee. The patients/participants provided their written informed consent to participate in this study.

## Author Contributions

TK, YU, TY, and SK designed the reported experiments, interpreted the results, and authored the manuscript. TK analyzed the data. All authors contributed to the article and approved the submitted version.

### Conflict of Interest

TK, YU, TY, and SK are employees of NTT Communication Science Laboratories, which is a basic-science research section of Nippon Telegraph and Telecommunication corporation (NTT). There is no pending patent involving the reported research. There are no products in development or marketed products to declare. Thus, the authors adheres to policies of Frontiers.
